# First Genome of Rock Lizard *Darevskia valentini* Involved in Formation of Several Parthenogenetic Species

**DOI:** 10.3390/genes13091569

**Published:** 2022-09-01

**Authors:** Sofia Ochkalova, Vitaly Korchagin, Andrey Vergun, Avel Urin, Danil Zilov, Sergei Ryakhovsky, Anastasiya Girnyk, Irena Martirosyan, Daria V. Zhernakova, Marine Arakelyan, Felix Danielyan, Sergei Kliver, Vladimir Brukhin, Aleksey Komissarov, Alexey Ryskov

**Affiliations:** 1 Laboratory of Genome Organization, Institute of Gene Biology of the Russian Academy of Sciences, Vavilova Str., 34/5, Moscow 119334, Russia; 2 Applied Genomics Laboratory, SCAMT Institute, ITMO University, Saint Petersburg 197101, Russia; 3 Department of Biochemistry, Molecular Biology and Genetics, Moscow Pedagogical State University, 1/1 M. Pirogovskaya Str., Moscow 119991, Russia; 4 Laboratory of Genomic Diversity, Center for Computer Technologies, ITMO University, St. Petersburg 197101, Russia; 5 Department of Zoology, Yerevan State University, 1 Alex Manoogian, Yerevan 0025, Armenia; 6 Department of the Diversity and Evolution of Genomes, Institute of Molecular and Cellular Biology SB RAS, 8/2 Acad. Lavrentiev Ave., Novosibirsk 630090, Russia; 7 Department of Plant Embryology & Reproductive Biology, Komarov Botanical Institute RAS, 2 Professor Popov Street, Saint-Petersburg 197376, Russia; 8 Laboratory of Molecular Systematics and Evolutionary Genetics, Florida Museum of Natural History Dickinson Hall Department of Natural History, Gainesvile, FL 32611, USA

**Keywords:** genome sequencing, de novo genome assembly, rock lizard, parthenogenesis, Lacertidae, *Hox* genes, microRNA

## Abstract

The extant reptiles are one of the most diverse clades among terrestrial vertebrates and one of a few groups with instances of parthenogenesis. Due to the hybrid origin of parthenogenetic species, reference genomes of the parental species as well as of the parthenogenetic progeny are indispensable to explore the genetic foundations of parthenogenetic reproduction. Here, we report on the first genome assembly of rock lizard *Darevskia valentini*, a paternal species for several parthenogenetic lineages. The novel genome was used in the reconstruction of the comprehensive phylogeny of Squamata inferred independently from 7369 trees of single-copy orthologs and a supermatrix of 378 conserved proteins. We also investigated *Hox* clusters, the loci that are often regarded as playing an important role in the speciation of animal groups with drastically diverse morphology. We demonstrated that *Hox* clusters of *D. valentini* are invaded with transposons and contain the *HoxC1* gene that has been considered to be lost in the amniote ancestor. This study provides confirmation for previous works and releases new genomic data that will contribute to future discoveries on the mechanisms of parthenogenesis as well as support comparative studies among reptiles.

## 1. Introduction

Non-avian reptiles are highly diverse and species-rich clade representing almost 11,000 out of more than 30,000 living amniote species [1,2]. It is one of the rare animal groups that acquired parthenogenesis, a form of asexual reproduction in which embryos develop from unfertilized eggs. Among vertebrates, it was first described in the lizard genus *Darevskia* inhabiting the Caucasus Mountains [3]. In the genus, as in most known instances, parthenogenetic species originated from interspecific hybridization between closely related bisexual species [4,5]. In total, the genus *Darevskia* includes 29 bisexual and 7 diploid parthenogenetic species of hybrid origin [2]. Yet only four parental bisexual species are known to be involved in the successful formation of parthenogenetic lineages: *D. raddei* and *D. mixta* being maternal species and *D. valentini* and *D. portschinskii* being paternal species [6,7]. Among seven parthenogenetic lineages four, namely *D. unisexualis*, *D. uzzelli*, *D. sapphirina*, and *D. bendimahiensis*, have the same maternal (*D. raddei*) and paternal (*D. valentini*) species [8]. It remains unknown which genetic or genomic factors play a key role in the generation and persistence of parthenogenetic organisms, and whether any structural (genomic) or/and functional (transcriptomic) changes in hybrids are associated with transition from sexual to clonal reproduction.

To further elucidate the role of interspecific hybridization in the formation of parthenogenetic organisms and study diversity and evolution of reptilian lineage, new data is needed on the genomics and transcriptomics of parthenogenetic species and their parental species. Therefore, we plan to sequence genomes and study genome content of the father–mother–progeny species trio of *D. valentini*, *D. raddei* and *D. unisexualis*, which has not been done before for any obligate parthenogenetic vertebrate.

Another intriguing aspect of reptilian diversity is repetitive genome sequences, especially mobile elements, which have repeatedly been associated with increased speciation rate and phenotypic richness [9,10,11,12]. It has been shown that transposition of mobile elements causes chromosomal rearrangements as well as segmental duplications and insertions/deletions relocating genes, regulatory elements, or their fragments [13]. Altogether, these alterations may constitute a fuel that powers a rapid evolution of populations under natural selection. Furthermore, transposable elements can affect gene regulation for introducing phenotypic novelties in the lineage and enhancing diversity [14,15]. Yet the entire scope of evolutionary mechanisms involving mobile DNA are largely “terra incognita”, particularly in non-model species.

It is hypothesized that speciation may be potentiated by adaptive alterations within the *Hox* gene clusters [10,11,12]. *Hox* genes encode transcription factors that are vital for coordination of embryonic development [16]. Two rounds of genome duplication resulted in formation of four *Hox* clusters (*HoxA*, *HoxB*, *HoxC*, and *HoxD*) from the ancestral set of *Hox* genes early in the vertebrate lineage [17,18]. Subsequently, functional redundancy has led to the loss of several *Hox* genes [19]. Despite this, gene order, coding sequence, as well as intergenic and tight clustered organization were believed to be maintained and conserved throughout amniote evolutionary history. However, comparison between *Hox* clusters of mouse, chicken, frog and green anole demonstrated atypical enlarged *Hox* clusters in the anole lizard with a massive accumulation of transposable elements in intergenic space [12]. A recent study showed that mobile elements accumulation correlates with alterations in *Hox* genes expression during development in *Anolis* lizards and can possibly contribute to their speciation [20].

Besides the homeobox genes themselves, metazoan *Hox* clusters contain microRNA genes, which are known to modulate *Hox* genes expression [21,22]. Generally, the prerequisite for the emergence of a new miRNA is a transcribed genomic sequence that form RNA hairpin structure capable of binding the miRNA complex. As RNA easily produces hairpin-like folds, the evolution of a novel miRNA gene seems to be more likely than the emergence of a new protein-coding gene [23]. While the *Hox* clusters that do not acquire new proteins over evolutionary epochs, miRNA genes can act as more fine-tuned regulators of developmental patterning, possibly reflecting peculiarities of morphogenesis of particular animal groups. This is supported by the fact that in the clusters there are known to be both highly conserved miRNA genes, namely *miR-10*, shared between arthropods and vertebrates, and *miR-196*, that are chordate-specific [24,25], and also more recently acquired miRNA genes. A well studied example is the *miR-615* gene, restricted to mammals. Unlike intergenic *miR-10* and *miR-196*, *miR-615* is nested within an intron of the *HoxC5* gene, which is characteristic of younger miRNA [26]. Intronic origin ensures transcription, and elimination of the need to evolve a separate promoter facilitates miRNA innovation [26,27]. Other important contributors to the evolution of younger, less conserved and lineage-specific miRNA genes are transposable elements [28,29]. In the human genome, hundreds of miRNA genes are proposed to be DNA transposon- or retrotransposon-derived [29,30]. Diversity of miRNA genes in non-model species is poorly studied, however it is reasonable to assume that clade-specific repeat family expansions may be followed by the emergence of new miRNA genes.

Thus, the investigation of the *Hox* clusters is of interest in terms of the evolution of its conserved protein-coding genes, miRNA genes, and mobile elements because they are likely to influence speciation and diversity. However, the available studies are mostly limited to model species.

**Figure 1:**
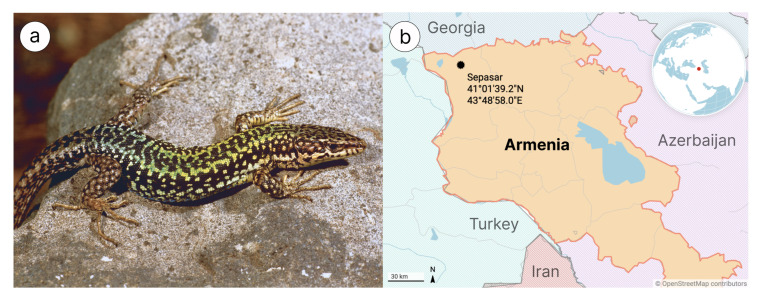
(**a**) Caucasian rock lizard *Darevskia valentini*, Armenia, from Arakelyan et al. [31]. (**b**) Map with DNA and RNA samples location (generated with Datawrapper).

In this paper, we first report on genome sequencing, assembly and annotation of *D. valentini* (Figure 1)—a paternal species for several parthenogenetic *Darevskia* lineages. Our main goal was to generate a reference genome assembly for parental species, which, along with the genomes of the second parental species and the F1 parthenogenetic hybrid, will be used for a future comprehensive study on parthenogenesis of hybrid origin. Besides, to get a more complete picture of evolution in *Hox* cluster organization and content of repetitive elements between mammals and reptiles, we compared this loci in human and *D. valentini* genomes. Considering a number of existing contradictions in evolutionary relationships within the diverse Squamata clade, we made an exhaustive phylogenomic reconstruction using two different approaches to obtain a robust species tree.

## 2. Materials and Methods

### 2.1. Samples Collection and Surgical Procedures

*Darevskia valentini* samples for DNA and RNA analysis were collected in 2019 and 2016, respectively, in Armenia outside of protected areas. This lizard species is not protected by CITES [https://cites.org/, 1 May 2021]. All individuals were hand-caught, and procedures for live-animal handling were approved by Yerevan State University strictly following ethical guidelines with capture permit Code 5/22.1/51,043 issued by the Ministry of Nature Protection of the Republic of Armenia for scientific studies. Blood samples of one individual (Sepasar population; 41°01${}^{\prime }$39.2${}^{\prime \prime }$ N 43°48${}^{\prime }$58.0${}^{\prime \prime }$ E, Figure 1) were taken from the tail vein and stored in 0.05 M EDTA buffers under +4 °C, and then this lizard was released. A single adult lizard was used to surgically collect tissues (liver, kidneys, brain, and heart) after decapitation. All tissue samples were stored at −20 °C in RNAlater® reagent according to the manufacturer’s recommended protocol (Qiagen, Dusseldorf, Germany) until they were transferred to Macrogen (Seoul, Korea) for RNA extraction, library preparation and total transcriptome sequencing. The study was approved by the Ethics Committee of Moscow State University (Permit Number: 24–01) and was conducted in strict accordance with ethical principles and scientific standards.

### 2.2. Extraction, Preparation and Sequencing of DNA Libraries

Total genomic DNA was isolated from lizard blood by using the standard phenol-chloroform extraction method with proteinase K and resuspended in TE buffer, pH 8.0. The samples were kept at +4 °C until they were processed. The DNA degradation and contamination were checked through agarose gel electrophoresis on 0.8% agarose gel followed by quantitation at 260 nm using Genesys® UV-Vis spectrophotometer (Thermo Fisher Scientific, Waltham, MA, USA). Afterwards, three DNA samples were shipped to Macrogen (Seoul, Korea) for genomic library preparation and sequencing. The 10x Chromium library was prepared according to the manufacturer’s instructions and sequenced on a HiSeq 2500 with a pair-end 150 bp reads.

### 2.3. Transcriptome Libraries Preparation and Sequencing

Total RNA was isolated from the tissues according to standard Trizol Tissue RNA Extraction protocol. After quality control, extracted RNAs were pooled together and sequenced. Strand-specific RNA sequencing was performed by Macrogen (Seoul, Korea) on Illumina HiSeq 2500 using TruSeq RNA Sample Prep Kit v2 for library preparation with a mean 101 bp read length.

### 2.4. Raw Data Preprocessing and Quality Control

RNA-seq reads underwent quality control and filtration. Quality assessment of all the sequencing data was performed using FastQC program [32]. Raw reads were trimmed with v2trim program (github.com/aglabx/Tools, accessed on 20 September 2021) and optical duplicates were removed with rmdup program (github.com/aglabx/Tools, accessed on 20 September 2021). Then, the reads were additionally cleaned from adapters by Cookiecutter [33], and next by Trimmomatic [34] with default parameters. We did not preprocess DNA reads as this step is included in the workflow of the assembler we used. We only run Jellyfish 2 [35] to calculate k-mer frequencies and Genomescope2 [36] to estimate genome size and rate of heterozygosity.

### 2.5. Genome Assembly and Quality Control

Raw DNA reads were assembled with Supernova 2 [37] with recommended parameters except maxreads equal to 485,333,333 (estimated from expected genome size 1.3 Gb and read length 150 bp according to Supernova tutorial). The accuracy of assembly was validated by examining Benchmarking Universal Single-Copy Orthologs (BUSCO v5.1 [38]) from the eukaryota_odb10 and sauropsida_odb10 databases that contain 255 and 7480 ultra-conserved protein families for eukaryotes and Reptilia, respectively. Assembly quality metrics for contiguity assessing were calculated using QUAST [39]. To detect possible contamination, we binned the assembled contigs with Metabat2 v.2.12 [40], and determined the taxonomic origin of the bins with CheckM [41] and BUSCO v5.1 (for possible bacterial and eukaryotic contamination, respectively).

### 2.6. Genome Annotation

#### 2.6.1. Repeats Annotation

To detect repetitive sequences in the genome assembly of *D. valentini*, we use de novo prediction with RepeatModeler2 [42]. Based on the obtained library, we processed the assembly with RepeatMasker v.4.1.1 (http://www.repeatmasker.org/, accessed on 20 September 2021) to identify and to mask repeats. Repeats for the human genome were extracted from the RepeatMasker output file derived from NCBI RefSeq (GCF_000001405.39_GRCh38.p13_rm.out).

#### 2.6.2. Gene Prediction and Functional Annotation

To perform gene structure annotations, we utilized the BRAKER2 pipeline [43] on the softmasked genome assembly. Training was supported by RNA-seq hints of *D. valentini* and alignment information from proteins of *Podarcis muralis*, which is closely related to *D. valentini*, and therefore their proteins are of close homology. To generate RNA hints, we aligned reads from transcriptome sequencing of *D. valentini* to its genome assembly with STAR v2.7.7a [44]. After gene prediction we applied the eggNOG-mapper v2 [45], which uses the eggNOG database [46] of protein orthologs to assign functions to predicted genes.

#### 2.6.3. *Hox* Clusters Annotation

For identification of genes from *Hox* clusters (A-D) in *D. valentini* genome we used functional gene annotation of our assembly produced by eggNOG-mapper. The *Hox* genes that we were unable to find in this way (*HoxC1*, *HoxC3*) were located by the alignment of the corresponding proteins of the gecko *Hemidactylus bowringii* (NCBI Accession ID: AEB32563.1) and *Latimeria menadoensis* (NCBI Accession ID: ACL81453.1) on the predicted proteins of *D. valentini* with BLASTP. After ensuring that the location on the scaffold of the best blast hits is consistent with the structure of the *Hox* cluster determined by the already discovered genes, they were added to the *Hox* genes dataset of *D. valentini*. Known miRNA genes were located by alignment of *Anolis carolinensis* miRNA hairpin sequences deposited to miRBase [47]. To visualize human *Hox* clusters and miRNA genes we used the genome annotation GCF_000001405.39 derived from NCBI RefSeq.

### 2.7. Phylogenomic Reconstruction

Phylogenetic relationships between *Darevskia valentini* and other species of Squamata were reconstructed using BUSCO Phylogenomics pipeline [48] that utilizes BUSCOs [38] to determine species phylogenies. The pipeline implements the following strategy: each group of ortholog genes is aligned with MUSCLE [49] and after trimming with trimAl [50] is processed by IQ-TREE [51] to generate independent ML phylogeny (supertree mode) or after trimming alignments are concatenated to generate a cumulative ML phylogeny (supermatrix mode).

We have used all representative genomes of squamates currently available in the National Center of Biotechnology Information (NCBI) genome database. Tuatara (*Sphenodon punctatus*) was set as an outgroup. For all the mentioned species and our genome assembly of *D. valentini* we find BUSCOs from the most specific available database sauropsida_odb10. We used both supertree and supermatrix approaches and inferred coalescent species trees with Astral v.5.7.5 [52].

## 3. Results and Discussion

### 3.1. Genome Assembly, Validation and Annotation

**Figure 2:**
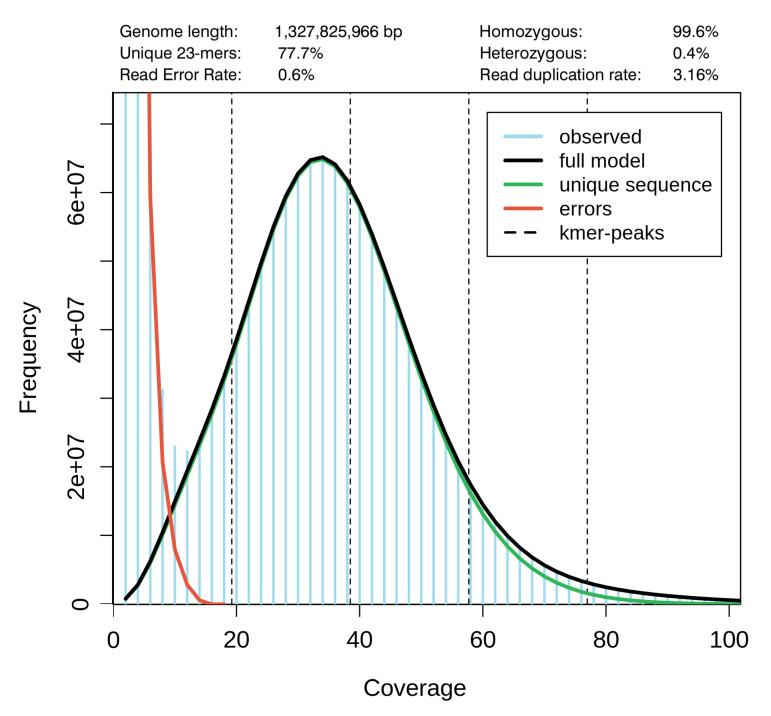
GenomeScope k-mer frequency plot for *Darevskia valentini*.

Using 10x linked reads library preparation with following Illumina sequencing we assembled a scaffold scale genome of 1.46 Gb size from a total of 180 Gb raw data, composed of 32,139 scaffolds with N50 equal to 3.94 Mb (refer to Table 1 for details). The size of the genome assembly was close to the k-mer based estimation (1.33 Gb, Figure 2). The 23-k-mer plot showed a one-peak distribution, indicating a low heterozygosity level.

To validate the correspondence between raw reads and the assembly, we mapped raw reads back to the assembly with BWA-MEM [53]. The 99.1% of reads were mapped back to the assembly; the remaining 0.9% of unmapped reads possibly arises from unassembled heterochromatic regions including centromeric regions.

We have assessed accuracy and completeness of the novel genome assembly of *D. valentini* using BUSCO v5.121. The approach is based on evolutionary principles and involves genome assembly screening for a defined set of conserved single-copy orthologs. Search for proteins from the general eukaryota-specific dataset (eukaryota_odb10, 255 gene families) and sauropsida-specific dataset (sauropsida_odb10, 7480 gene families) revealed that the assembly included a large percentage of full-length genes (97.3% and 87.8%, respectively; Table 2), indicating the genome sequence is accurate. To prevent the addition of foreign DNA into the assembly we binned the assembly and made further taxonomic assignment of bins with CheckM and BUSCO, that do not reveal any contamination.

In addition, the genome of *D. valentini* was predicted to contain a high number of repetitive DNA sequences, which comprise 41.5% of all genome length. Of these, half are of unknown type due to the absence of significant matches with the available repeat databases and are either lizard-specific and not included in Repbase and Dfam repeat libraries or too degenerated to be identified. Among classified repeats, most abundant are non-LTR retroelements: LINEs (12.9% of sequences) with 8.8%, 2.2%, 1.6% from L2/CR1/Rex, RTE/Bov-B, L1/CIN4 families, respectively, and SINEs (1.8% of sequences). Another common category is DNA transposons (2.9% of sequences), of which 2.3% are hAT-transposons and 0.5% are Tc1 family. LTR retroelements comprise about 1% of genome length. Satellites, together with microsatellites and low-complexity sequences, occupy about 1.7% of the total DNA.

**Figure A1:**
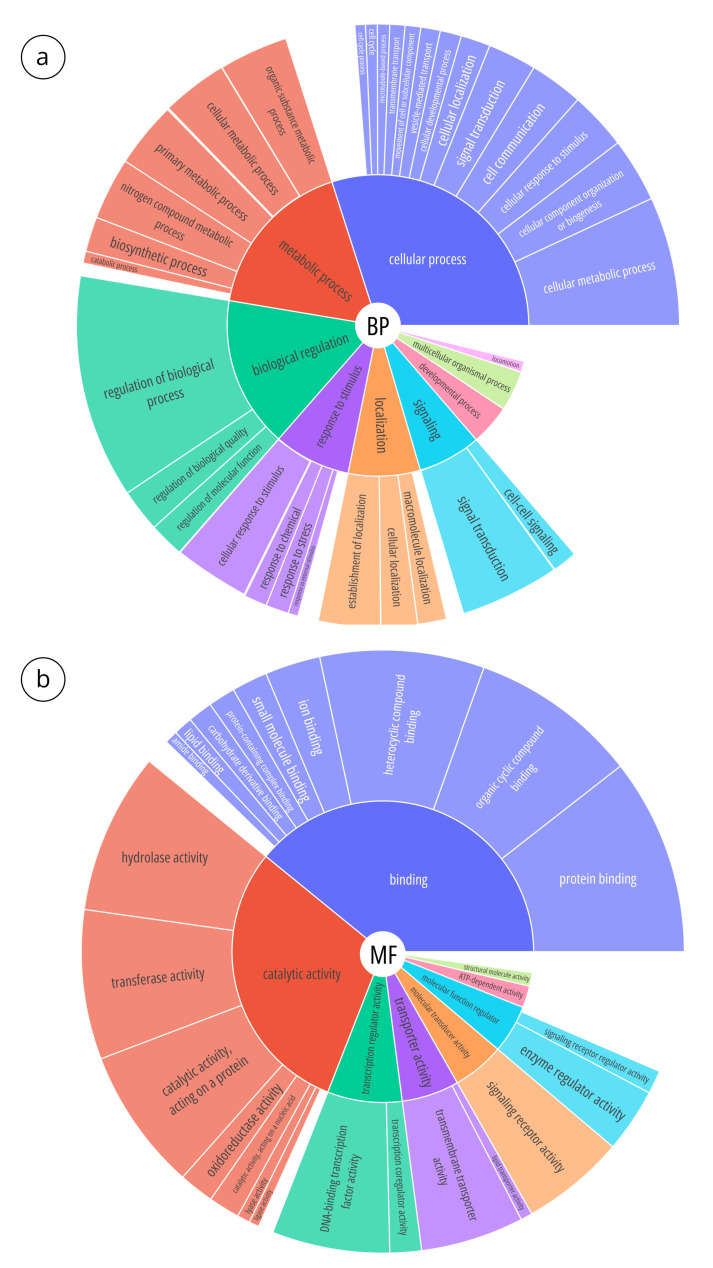
Functional classification of genes, according to Gene Ontology terms from EggNOG-mapper annotation of *Darevskia valentini*. (**a**)—Biological process, (**b**)—Molecular function. For better visibility, sparsely represented terms (<100 genes) are not shown.

Transcriptome sequencing generated 5.8 Gb of raw data that was used for gene prediction. Altogether, the annotation yielded a set of 26,986 protein-coding genes with function assigned based on homology with EggNOG database. Functional classification of genes by assigned Gene Ontology terms is shown in Figure A1.

### 3.2. Hox Cluster Organisation in D. valentini

**Figure 3:**
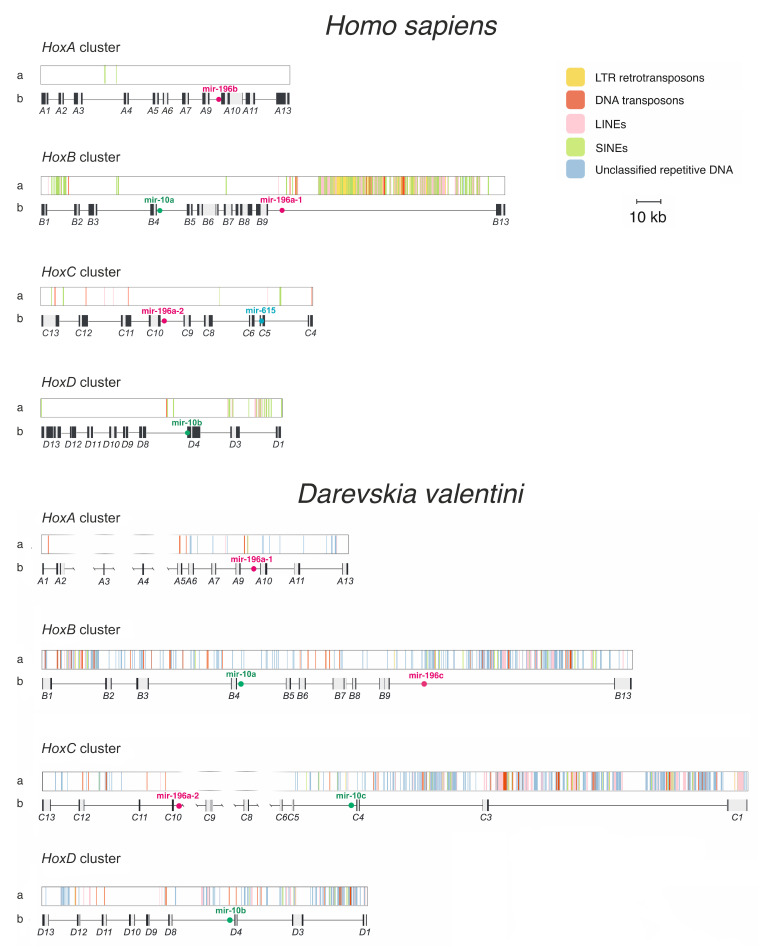
Comparison of *Hox* gene clusters (A–D) in *Darevskia valentini* and human genomes. (**a**) Mobile elements within each of the clusters: yellow—LTR retrotransposons, red—DNA transposons, pink—LINEs, green—SINEs, blue—unclassified repeat type. (**b**) *Hox* genes (grey—introns, black—exons) and miRNA genes. For several human genes, only coding DNA sequences (CDS) are visualized.

In order to better understand evolution of *Hox* clusters in squamates, we annotated *Hox* genes in the *D. valentini* genome assembly and investigated mobile elements present in the clusters as well as compared obtained results with the human *Hox* clusters organization and content (Figure 3).

In general, *D. valentini Hox* genes show a typical clustered organization in a single intact cluster structure, that was described earlier for vertebrates [54]. *HoxA* and *HoxC* are separated across multiple scaffolds due to incomplete assembly, which obscures their relative size between human and lizard. Gene arrangement is comparable, except for *HoxC3*, which is absent in mammal genomes [19]. Furthermore, we found in *HoxC* flanking gene *HoxC1*, which was considered to be lost in the amniote ancestor [19]. It is consistent with the previous findings revealing the exclusive presence and functional activity of this gene in some lizard species and confirms the suggestion that this gene is maintained in lizard lineage, in contrast to other amniotes [55].

In both genomes, length of the protein-coding and intronic sequences of *Hox* genes are generally comparable. A difference in exon sizes, which seem longer in human genome, arises from an incompleteness of the de novo *Darevskia* annotation, where actual coding DNA sequences (CDS) are treated as exons due to an inability to correctly identify untranslated regions (UTRs) in a novel genome without extrinsic evidence [56]. Although intergenic distances in *D. valentini* are larger than human counterparts, in general the difference is less distinct than previously described for green anole [12]. *Hox* clusters in the anole genome were reported to be significantly larger than in mammals, up to 2.5-fold (*HoxD*). At the same time, we can observe that in *D. valentini* the size difference does not exceed 1.4-fold (*HoxD*).

The differences in cluster sizes, intergenic and intronic region lengths between two species probably arises from massive accumulation of interspersed repeats in lizard *Hox* cluster, not observed in the human genome and usually not tolerated in these loci [57,58]. In the rock lizard’s *Hox* clusters average content of predicted interspersed elements is about 15%, compared to 2% in human loci.

Apart from content and distribution of interspersed repeats, their nature also differs greatly between species. In the human *Hox* clusters, high density of transposons is observed only in an atypically large intergenic space between *HoxB9* and *HoxB13*, as well as smaller regions *HoxB1*-*HoxB2* and *HoxD1*-*HoxD3*. About half of the interspersed repeats are Alu family SINEs, others are mainly L1 or L2 LINEs and LTR retrotransposons from ERVL family.

Compared to human, *D. valentini Hox* loci displays high repeat frequency not only at the extremities, but along the entire extent of the clusters. Perhaps the assembly failure in the *HoxA* and *HoxC* clusters is a result of the repeat accumulation in these regions. On average 55% of repeats found in *D. valentini Hox* clusters are of unknown type. Classified mobile DNA mostly contains LINEs (CR1, L1, L2, RTE, Penelope) and hAT DNA transposons. Surprisingly, many *Hox* genes have hAT-transposon inserted inside their introns or adjacent regions. Lizard *HoxC1* contains an intron almost 9 kb in size, which is larger than any human intron. It contains a long LINE-2 transposon along with other repeat-derived sequences altogether constituting half the intron length.

Previously, the green anole genome first evidenced the capability of accumulating transposons in the amniote *Hox* loci, yet genomes of other squamates were required to confirm the generality of this property for the entire clade [12]. We show that another lizard species, *D. valentini*, from the Lacertidae family shares this feature. As lacertid lizards are older than Iguanidae including *Anolis* genus, we hypothesize that paradigm shift in *Hox* clusters’ organization occurred no later than in early history of Squamata in a common ancestor of Episquamata. At the same time, the clusters’ enlargement in anole is substantially stronger than what we observe in *Darevskia*, which may indicate varying degrees of tolerance to repeats in *Hox* clusters among species.

The presence of repeats in the *Hox* cluster suggests that their insertions have no detrimental effect or are even beneficial for the host organism. Loosened constraints of cluster size may stem from the different ways in which clusters function in lizards and humans, which in the former case relies less on a tight clustering than in the latter. One opinion is that *Hox* genes are kept together due to shared regulatory sequences or overlapping transcriptional units [59]. Therefore, modifications in a system of *cis*-regulatory elements in the lizard or reptile lineage could result in relaxed constraints. A different set of genes containing *HoxC1* and *HoxC3* in lizards also favors a different mechanism of regulation.

The benefit from the presence of repeats may be conditioned by their contribution to the phenotype diversity. The most prevalent repetitive elements identified in *D. valentini Hox* loci are LINEs, SINEs, and DNA hAT-transposons, which can be a rich source of regulatory variations [60,61]. The likely candidates are hAT-transposons embedded in introns or regions adjacent to genes, which are found widely in the *Darevskia* lizard clusters. Transposable elements insertion can introduce new transcriptional boundary elements, and this way affects the gene expression [62]. Moreover, there are many transposon-derived miRNAs that are crucial for post-transcriptional expression regulation [63]. Yet, as was found in diverse mammalian cell types [64,65], some transposable elements included in genes, such as SINEs and LINEs [66,67], might participate in the splicing of the precursor of mRNA (pre-mRNA) and in the formation of non-coding circular RNAs [68,69], that function as efficient miRNA sponges [70], thus diminishing the effect of miRNA on transcriptional and post-transcriptional levels of regulation of gene expression [71]. The functionality of transposons in the *Hox* cluster became more plausible with the discovery of a correlation between the accumulation of mobile elements and the level of *Hox* genes’ expression during development in *Anolis* lizards [20]. However, so far, it is unclear what part of transposons remains functional, since most of them are considerably degenerated.

The miRNA genes examined were limited to the green anole genes previously published in the miRBase by Lyson et al. [72], because we lack small RNA sequencing data for *D. valentini*. As expected, *D. valentini Hox* clusters encode known conserved miRNA genes *mir-10a*, *mir-10b*, *mir-196a*, located between *HoxB4-HoxB5*, *HoxD4-HoxD8* and *HoxC9-HoxC10*, respectively, that were previously described in the human, mouse and zebrafish *Hox* clusters. While the human *HoxC* cluster has mammalian-specific *mir-615* miRNA, *D. valentini* has a *mir-10c* variant of *mir-10* family of miRNA in intergenic spacer of *HoxC4* and *HoxC5*. According to the data of miRBase, *mir-10c* is also present in the genomes of green anole, alligator (*Alligator mississippiensis*), king cobra (*Ophiophagus hannah*) and turtle (*Chrysemys picta*), but absent in chicken and mammals. Apparently, this miRNA gene is restricted to reptiles. Besides, *D. valentini* has another variant of *mir-196* miRNA between *HoxB9* and *HoxB13*, *mir-196c*, that has also been found in genomes of green anole and two snake species (*Ophiophagus hannah, Python bivittatus*) and may be unique for squamates. Additionally, we have not found *mir-196b* in *D. valentini Hox* loci. Contrary to the expectations, none of observed miRNA genes are transposon-derived or of intronic origin, instead all found miRNA genes are variants of conserved *mir-196* and *mir-10* families. It emphasizes the conservative nature of the *Hox* loci structure, which extends beyond protein-coding genes to regulatory non-coding RNA genes as well.

### 3.3. Phylogenomic Reconstruction of Squamate Phylogeny

**Figure 4:**
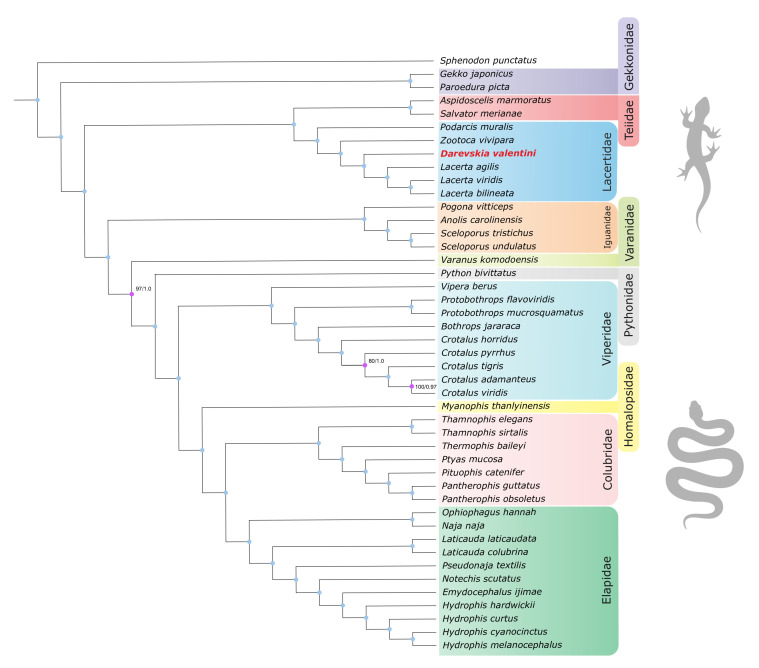
Coalescent species tree of lacertid lizards inferred from 7369 BUSCO maximum likelihood phylogenies. Branch support values were measured as bootstrap support (concatenation approach) and local posterior probabilities (coalescent tree approach). If not stated, the value is equal to 100/1.0 (bootstrap/posterior probability).

Based on the available whole-genome assemblies of Squamata and novel genome assembly of *D. valentini*, we have reconstructed evolutionary relationships inside the clade (Figure 4). Phylogenomic analysis was performed on the extensive dataset of protein sequences of single-copy orthologs from the BUSCO sauropsida_db10 database using the maximum likelihood (ML) method. We conducted two separate phylogenomic estimates with separate concatenation and coalescent species tree analyses that yielded similar tree topology with high branch support. The supermatrix was composed of 378 conserved proteins with a total length of 196,897 amino acids and the species tree was inferred from 7369 individual trees of protein orthologs.

The monophyly of all included subfamilies and tribes was unambiguously recovered. Early branches resolved with a full accordance with generally accepted phylogeny of squamates. Geckos are placed near the root of the tree.

Clade of Lacertidae is in agreement with the phylogeny suggested recently by Garcia-Porta et al. [73] that was based on transcriptome data and mitochondrial genes. The monophyly of the clade containing genera *Lacerta* and *Darevskia*, represented by the first genome of *D. valentini* we assembled, has been recovered. Our analysis also confirms that genus *Podarcis* is a sister group to other Lacertini, which was reported to be controversial among previous studies [73,74].

In general, reconstructed tree topology for suborder of Serpentes is congruent with the trees obtained recently both with a broad clade sampling based on 40 nuclear and 12 mitochondrial loci and phylogeny for limited number of species estimated from 40,000 genes [75,76]. However, we clarify relationships inside the genus *Crotalus*, that was not included in phylogenetic analysis with a sufficient number of loci before. Additionally, according to our findings the family Homalopsidae is an external group in relation to the clade including Colubridae and Elapidae, in previous studies this node was not resolved [74]. Besides, the genus *Thamnophis* is a sister clade to all other Colubridae, before this node had low support due to a small sampling of loci [74].

Sister relationships of *Ophiophagus* and *Naja*, and the consecutive nesting in relation to them of *Laticauda*, *Pseudonaja*, *Notechis*, *Emydocephalus*, and *Hydrophis*, were first validated using a sufficient set of markers for phylogenetic inference. The position of other families and topology of their clades are in absolute agreement with the earlier data supported by a smaller [74,76] or larger number of loci in the analysis [73,75].

## 4. Conclusions

In this paper, we present a de novo scaffold-scale genome assembly of rock lizard *D. valentini* generated with short-read sequencing. Evaluation of the assembly quality showed high values of main metrics: N50 of 3.94 Mb and 87.8% of complete BUSCO genes from sauropsida-specific dataset. Neither bacterial nor eukaryotic contamination was detected. Thus, we have succeeded in performing adequate and representative genome assembly of *D. valentini*, which is a reliable starting point for annotation and subsequent genome analysis.

Annotation of *Hox* clusters in *D. valentini* showed four intact gene clusters with 41 *Hox* genes in total, one more than the typical reptilian repertoire due to the presence of *HoxC1*. This observation supports the assumption that a *Hox* cluster structure of the amniote common ancestor is preserved in the lizard lineage [55].

While the gene order and coding sequences of the *Hox* genes are conserved in both human and *Darevskia* lizards, the lengths of the intergenic regions and the overall size of the clusters differ. The increased size of the clusters in *D. valentini* is probably a consequence of a massive accumulation of transposons.

The search of the miRNA database of known green anole’s miRNAs did not reveal any novel genes that emerged in the reptilian *Hox* clusters; however, variants of *mir-196* and *mir-10* were found that appear to be specific to reptiles or squamates.

A novel genome of *D. valentini* was also used by us in phylogenomic reconstruction of evolutionary relationships inside Squamata. Our phylogenetic estimate covered all squamates with a sequenced genome deposited to NCBI, 44 species in total, and relied both on supermatrix and species tree methods that yielded congruent trees with high branch support. Obtained results validate previous studies with limited taxon sampling, insufficient number of loci or based on a less accurate phylogenetic method as well as propose new relationships at the level of species and families. The updated data will be a good basis for future comparative studies within Squamata.

## Figures and Tables

**Table 1:** Genome assembly and annotation statistics.

Characteristics	Value
Genome length (bp)	1,456,729,600
Number of scaffolds	32,139
Scaffold N50 (bp)	3,939,878
Scaffold L50 (bp)	80
%N	3.73
GC content	43.96%

**Table 2:** Result of the BUSCO screening on the genome assembly of *Darevskia valentini*.

Category of BUSCOs	eukaryota_odb10	sauropsida_odb10
Number of protein groups in the database	255	7480
Complete	247 (96.9%)	6552 (87.6%)
Complete and single-copy	241 (94.5%)	6313 (84.4%)
Complete and duplicated	6 (2.4%)	239 (3.2%)
Fragmented	6 (2.4%)	301 (4.0%)
Missing	2 (0.7%)	627 (8.4%)

## Data Availability

Assembled genome contigs of *D. valentini* were released in the NCBI Assembly database as GCA_024498535.1. Bioproject PRJNA327916 and SRA SRX12402752 (high coverage WGS), SRX12402757 and SRX12402756 (low coverage WGS), and SRX12402758 (10x data).

## References

[1] Tollis M., Hutchins E.D., Kusumi K. (2014). Reptile genomes open the frontier for comparative analysis of amniote development and regeneration. Int. J. Dev. Biol..

[2] Uetz P., Freed P., Aguilar R., Hošek J. (2021). The Reptile Database. www.reptile-database.org.

[3] Arribas O.J. (1999). Phylogeny and Relationships of the Mountain Lizards of Europe and Near East (*Archaeolacerta* Mertens, 1921, *sensu lato*) and Their Relationships Among the Eurasian Lacertid Radiation. Russ. J. Herpetol..

[4] Darevsky I., Kupriyanova L., Uzzell T. (1985). Parthenogenesis in Reptiles. Biology of Reptilia.

[5] Neaves W.B., Baumann P. (2011). Unisexual reproduction among vertebrates. Trends Genet..

[6] Murphy R.W., Fu J., Macculloch R.D., Darevsky I.S., Kupriyanova L.A. (2000). A fine line between sex and unisexuality: The phylogenetic constraints on parthenogenesis in lacertid lizards. Zool. J. Linn. Soc..

[7] Fu J., Murphy R., Darevsky I. (1997). Toward the phylogeny of caucasian rock lizards: Implications from mitochondrial DNA gene sequences (Reptilia: Lacertidae). Zool. J. Linn. Soc..

[8] Schmidtler J., Eiselt J., Darevsky I. (1994). Untersuchungen an Felseidechsen (Lacerta saxicola Gruppe) in der östlichen Türkei: 3. Zwei neue parthenogenetische Arten. Salamandra.

[9] Ahmad S., Singchat W., Jehangir M., Panthum T., Srikulnath K. (2020). Consequence of Paradigm Shift with Repeat Landscapes in Reptiles: Powerful Facilitators of Chromosomal Rearrangements for Diversity and Evolution. Genes.

[10] Voss S.R., Putta S., Walker J.A., Smith J.J., Maki N., Tsonis P.A. (2013). Salamander Hox clusters contain repetitive DNA and expanded non-coding regions: A typical Hoxstructure for non-mammalian tetrapod vertebrates?. Hum. Genom..

[11] Feiner N. (2016). Accumulation of transposable elements in *Hox* gene clusters during adaptive radiation of *Anolis* lizards. Proc. R. Soc. B Biol. Sci..

[12] Di-Poï N., Montoya-Burgos J.I., Duboule D. (2009). Atypical relaxation of structural constraints in *Hox* gene clusters of the green anole lizard. Genome Res..

[13] Kazazian H.H. (2004). Mobile Elements: Drivers of Genome Evolution. Science.

[14] Warren I.A., Naville M., Chalopin D., Levin P., Berger C.S., Galiana D., Volff J.N. (2015). Evolutionary impact of transposable elements on genomic diversity and lineage-specific innovation in vertebrates. Chromosome Res..

[15] Trizzino M., Park Y., Holsbach-Beltrame M., Aracena K., Mika K., Caliskan M., Perry G.H., Lynch V.J., Brown C.D. (2017). Transposable elements are the primary source of novelty in primate gene regulation. Genome Res..

[16] Duboule D. (1994). Guidebook to the Homeobox Genes.

[17] Holland P.W., Garcia-Fernàndez J., Williams N.A., Sidow A. (1994). Gene duplications and the origins of vertebrate development. Dev. Suppl..

[18] Lemons D., McGinnis W. (2006). Genomic evolution of Hox gene clusters. Science.

[19] Liang D., Wu R., Geng J., Wang C., Zhang P. (2011). A general scenario of Hox gene inventory variation among major sarcopterygian lineages. BMC Evol. Biol..

[20] Feiner N. (2019). Evolutionary lability in *Hox* cluster structure and gene expression in *Anolis* lizards. Evol. Lett..

[21] Lempradl A., Ringrose L. (2008). How does noncoding transcription regulate Hox genes?. BioEssays.

[22] Petruk S., Sedkov Y., Brock H.W., Mazo A. (2007). A Model for Initiation of Mosaic HOX Gene Expression Patterns by Non-Coding RNAs in Early Embryos. RNA Biol..

[23] Chen K., Rajewsky N. (2007). The evolution of gene regulation by transcription factors and microRNAs. Nat. Rev. Genet..

[24] Tanzer A., Amemiya C.T., Kim C.B., Stadler P.F. (2005). Evolution of microRNAs located withinHox gene clusters. J. Exp. Zool. Part Mol. Dev. Evol..

[25] Candiani S. (2012). Focus on miRNAs evolution: A perspective from amphioxus. Briefings Funct. Genom..

[26] Campo-Paysaa F., Sémon M., Cameron R.A., Peterson K.J., Schubert M. (2011). microRNA complements in deuterostomes: Origin and evolution of microRNAs: miRNA origins and evolution. Evol. Dev..

[27] Meunier J., Lemoine F., Soumillon M., Liechti A., Weier M., Guschanski K., Hu H., Khaitovich P., Kaessmann H. (2013). Birth and expression evolution of mammalian microRNA genes. Genome Res..

[28] Piriyapongsa J., Mariño-Ramírez L., Jordan I.K. (2007). Origin and Evolution of Human microRNAs from Transposable Elements. Genetics.

[29] Yuan Z., Sun X., Liu H., Xie J. (2011). MicroRNA Genes Derived from Repetitive Elements and Expanded by Segmental Duplication Events in Mammalian Genomes. PLoS ONE.

[30] Friedländer M.R., Mackowiak S.D., Li N., Chen W., Rajewsky N. (2012). miRDeep2 accurately identifies known and hundreds of novel microRNA genes in seven animal clades. Nucleic Acids Res..

[31] Arakelyan M., Danielyan F., Corti C., Sindaco R., Leviton A.E., Vasilyan D. (2011). Herpetofauna of Armenia and Nagorno-Karabakh.

[32] Andrews S. FASTQC. A Quality Control Tool for High Throughput Sequence Data 2010. https://github.com/s-andrews/FastQC.

[33] Starostina E., Tamazian G., Dobrynin P., O’Brien S., Komissarov A. (2015). Cookiecutter: A tool for kmer-based read filtering and extraction. bioRxiv.

[34] Bolger A.M., Lohse M., Usadel B. (2014). Trimmomatic: A flexible trimmer for Illumina sequence data. Bioinformatics.

[35] Marçais G., Kingsford C. (2011). A fast, lock-free approach for efficient parallel counting of occurrences of k-mers. Bioinformatics.

[36] Ranallo-Benavidez T.R., Jaron K.S., Schatz M.C. (2020). GenomeScope 2.0 and Smudgeplot for reference-free profiling of polyploid genomes. Nat. Commun..

[37] Weisenfeld N.I., Kumar V., Shah P., Church D.M., Jaffe D.B. (2017). Direct determination of diploid genome sequences. Genome Res..

[38] Seppey M., Manni M., Zdobnov E.M., Kollmar M. (2019). BUSCO: Assessing Genome Assembly and Annotation Completeness. Gene Prediction.

[39] Gurevich A., Saveliev V., Vyahhi N., Tesler G. (2013). QUAST: Quality assessment tool for genome assemblies. Bioinformatics.

[40] Kang D.D., Li F., Kirton E., Thomas A., Egan R., An H., Wang Z. (2019). MetaBAT 2: An adaptive binning algorithm for robust and efficient genome reconstruction from metagenome assemblies. PeerJ.

[41] Parks D.H., Imelfort M., Skennerton C.T., Hugenholtz P., Tyson G.W. (2015). CheckM: Assessing the quality of microbial genomes recovered from isolates, single cells, and metagenomes. Genome Res..

[42] Flynn J.M., Hubley R., Goubert C., Rosen J., Clark A.G., Feschotte C., Smit A.F. (2020). RepeatModeler2 for automated genomic discovery of transposable element families. Proc. Natl. Acad. Sci. USA.

[43] Brůna T., Hoff K.J., Lomsadze A., Stanke M., Borodovsky M. (2021). BRAKER2: Automatic eukaryotic genome annotation with GeneMark-EP+ and AUGUSTUS supported by a protein database. NAR Genom. Bioinform..

[44] Dobin A., Davis C.A., Schlesinger F., Drenkow J., Zaleski C., Jha S., Batut P., Chaisson M., Gingeras T.R. (2013). STAR: Ultrafast universal RNA-seq aligner. Bioinformatics.

[45] Huerta-Cepas J., Forslund K., Coelho L.P., Szklarczyk D., Jensen L.J., von Mering C., Bork P. (2017). Fast Genome-Wide Functional Annotation through Orthology Assignment by eggNOG-Mapper. Mol. Biol. Evol..

[46] Huerta-Cepas J., Szklarczyk D., Heller D., Hernández-Plaza A., Forslund S.K., Cook H., Mende D.R., Letunic I., Rattei T., Jensen L. (2019). eggNOG 5.0: A hierarchical, functionally and phylogenetically annotated orthology resource based on 5090 organisms and 2502 viruses. Nucleic Acids Res..

[47] Kozomara A., Birgaoanu M., Griffiths-Jones S. (2019). miRBase: From microRNA sequences to function. Nucleic Acids Res..

[48] McGowan J., Fitzpatrick D.A. (2020). Recent advances in oomycete genomics. Advances in Genetics.

[49] Edgar R.C. (2004). MUSCLE: Multiple sequence alignment with high accuracy and high throughput. Nucleic Acids Res..

[50] Capella-Gutiérrez S., Silla-Martínez J.M., Gabaldón T. (2009). trimAl: A tool for automated alignment trimming in large-scale phylogenetic analyses. Bioinformatics.

[51] Nguyen L.T., Schmidt H.A., von Haeseler A., Minh B.Q. (2015). IQ-TREE: A Fast and Effective Stochastic Algorithm for Estimating Maximum-Likelihood Phylogenies. Mol. Biol. Evol..

[52] Zhang C., Rabiee M., Sayyari E., Mirarab S. (2018). ASTRAL-III: Polynomial time species tree reconstruction from partially resolved gene trees. BMC Bioinform..

[53] Li H., Durbin R. (2009). Fast and accurate short read alignment with Burrows-Wheeler transform. Bioinformatics.

[54] Akam M. (1989). Hox and HOM: Homologous gene clusters in insects and vertebrates. Cell.

[55] Feiner N., Wood N.J. (2019). Lizards possess the most complete tetrapod Hox gene repertoire despite pervasive structural changes in Hox clusters. Evol. Dev..

[56] Yandell M., Ence D. (2012). A beginner’s guide to eukaryotic genome annotation. Nat. Rev. Genet..

[57] Amemiya C.T., Prohaska S.J., Hill-Force A., Cook A., Wasserscheid J., Ferrier D.E., Pascual-Anaya J., Garcia-Fernàndez J., Dewar K., Stadler P.F. (2008). The amphioxus Hox cluster: Characterization, comparative genomics, and evolution. J. Exp. Zool. Part B Mol. Dev. Evol..

[58] Fried C., Prohaska S.J., Stadler P.F. (2004). Exclusion of repetitive DNA elements from gnathostome Hox clusters. J. Exp. Zool..

[59] Kikuta H., Fredman D., Rinkwitz S., Lenhard B., Becker T.S. (2007). Retroviral enhancer detection insertions in zebrafish combined with comparative genomics reveal genomic regulatory blocks—A fundamental feature of vertebrate genomes. Genome Biol..

[60] Wicker T., Sabot F., Hua-Van A., Bennetzen J.L., Capy P., Chalhoub B., Flavell A., Leroy P., Morgante M., Panaud O. (2007). A unified classification system for eukaryotic transposable elements. Nat. Rev. Genet..

[61] Kojima K.K. (2019). Structural and sequence diversity of eukaryotic transposable elements. Genes Genet. Syst..

[62] Sundaram V., Wysocka J. (2020). Transposable elements as a potent source of diverse *cis* -regulatory sequences in mammalian genomes. Philos. Trans. R. Soc. B Biol. Sci..

[63] Petri R., Brattås P.L., Sharma Y., Jönsson M.E., Pircs K., Bengzon J., Jakobsson J. (2019). LINE-2 transposable elements are a source of functional human microRNAs and target sites. PLoS Genet..

[64] Salzman J., Gawad C., Wang P.L., Lacayo N., Brown P.O. (2012). Circular RNAs Are the Predominant Transcript Isoform from Hundreds of Human Genes in Diverse Cell Types. PLoS ONE.

[65] Salzman J., Chen R.E., Olsen M.N., Wang P.L., Brown P.O. (2013). Cell-Type Specific Features of Circular RNA Expression. PLoS Genet..

[66] Wilusz J.E. (2015). Repetitive elements regulate circular RNA biogenesis. Mob. Genet. Elem..

[67] Jeck W.R., Sorrentino J.A., Wang K., Slevin M.K., Burd C.E., Liu J., Marzluff W.F., Sharpless N.E. (2013). Circular RNAs are abundant, conserved, and associated with ALU repeats. RNA.

[68] Ashwal-Fluss R., Meyer M., Pamudurti N., Ivanov A., Bartok O., Hanan M., Evantal N., Memczak S., Rajewsky N., Kadener S. (2014). circRNA Biogenesis Competes with Pre-mRNA Splicing. Mol. Cell.

[69] Starke S., Jost I., Rossbach O., Schneider T., Schreiner S., Hung L.H., Bindereif A. (2015). Exon Circularization Requires Canonical Splice Signals. Cell Rep..

[70] Hansen T.B., Jensen T.I., Clausen B.H., Bramsen J.B., Finsen B., Damgaard C.K., Kjems J. (2013). Natural RNA circles function as efficient microRNA sponges. Nature.

[71] Filippenkov I.B., Kalinichenko E.O., Limborska S.A., Dergunova L.V. (2017). Circular RNAs—One of the enigmas of the brain. Neurogenetics.

[72] Lyson T.R., Sperling E.A., Heimberg A.M., Gauthier J.A., King B.L., Peterson K.J. (2012). MicroRNAs support a turtle + lizard clade. Biol. Lett..

[73] Garcia-Porta J., Irisarri I., Kirchner M., Rodríguez A., Kirchhof S., Brown J.L., MacLeod A., Turner A.P., Ahmadzadeh F., Albaladejo G. (2019). Environmental temperatures shape thermal physiology as well as diversification and genome-wide substitution rates in lizards. Nat. Commun..

[74] Pyron R.A., Burbrink F.T., Wiens J.J. (2013). A phylogeny and revised classification of Squamata, including 4161 species of lizards and snakes. BMC Evol. Biol..

[75] Tu N., Liang D., Zhang P. (2020). Whole-exome sequencing and genome-wide evolutionary analyses identify novel candidate genes associated with infrared perception in pit vipers. Sci. Rep..

[76] Klein C.G., Pisani D., Field D.J., Lakin R., Wills M.A., Longrich N.R. (2021). Evolution and dispersal of snakes across the Cretaceous-Paleogene mass extinction. Nat. Commun..

